# Examination of Intermolecular Forces Influencing Headspace Analysis of Biological Samples

**DOI:** 10.3390/metabo15030183

**Published:** 2025-03-09

**Authors:** Young Eun Lee, Bruce A. Kimball

**Affiliations:** Monell Chemical Senses Center, Philadelphia, PA 19104, USA; bkimball@monell.org

**Keywords:** headspace analysis, SPME, volatile metabolites, GC-MS, sample matrix effects

## Abstract

Headspace analysis is an effective method for assessing the concentrations of volatile and semi-volatile metabolites in biological samples. In particular, solid-phase microextraction (SPME) is an efficient tool for headspace analyses. Metabolites present in the sample are the typical targets of headspace analysis (rather than the vapor phase concentration) for making measurements on sample donors (e.g., biomarkers of health or disease). Accordingly, intermolecular forces between metabolites and matrix may prevent a complete profile of the metabolite composition in the biosamples from being revealed. To assess sources of such interactions, several volatile compounds in various sample mediums were examined. Small volatile metabolites typical of human biosamples were the volatile compounds selected for this study. Test media included lipid or serum solution to simulate biological samples commonly encouraged in biomarker discovery. Headspace concentrations of volatile analytes were compared using solid-phase microextraction gas chromatography-mass spectrometry (SPME-GC-MS). Observed levels of metabolites in headspace varied among the different media, despite being fortified at equal concentrations in the samples. Overall, lower headspace responses were observed in samples containing proteins or lipids. It was found that these strong intermolecular interactions arose from irreversible chemical bonds between the volatile molecules and component of the sample matrix. However, headspace responses could be maximized when the analysis was performed at temperatures ranging from 60 to 70 °C. Furthermore, normalization of peak responses to an internal standard did not always account for these interactions.

## 1. Introduction

Complex living organisms, such as humans and animals, consist of billions of cells generating a wide variety of metabolites, from macromolecules (e.g., proteins) to small molecules (e.g., acids and ketones) as products of countless chemical reactions [1,2]. A small portion of these small metabolites are volatile in nature. The study of volatile metabolites, while encompassing all living systems, has particularly focused on human metabolites, as it can enhance our understanding of biological processes within the human body in real time [3,4]. Volatile metabolites emitted from the human body are complex mixtures influenced by both endogenous and exogenous factors. While the exact origin of these volatile metabolites has yet to be fully understood, several pathways contribute to their production. They arise from both normal and abnormal metabolic processes occurring within the body [5,6,7,8], and are emitted in various forms, including skin emanations, urine, saliva, feces, and blood [9,10].

Volatile metabolites comprise a chemically diverse group of small organic compounds, generally with a molecular weight in the range of 50–200 Daltons. They exhibit significant vapor pressure under ambient conditions, contributing to distinctive odors [11]. If studies using trained animal biosensors (e.g., dogs) are any indication [12,13,14], volatile metabolites can serve as chemical indicators for assessing human health status in a non-invasive manner [15,16,17]. While various technologies are employed to detect volatile metabolites, GC-MS is the most commonly used. Headspace sampling with SPME is highly effective for the qualitative or quantitative analysis of volatile compounds, offering minimal extraction steps and reducing column contamination from sample sources [18]. SPME, invented in the early nineties, enables the solventless extraction of volatile analytes from a wide range of matrices [19,20]. It functions as a three-phase system, involving two key equilibria: (1) equilibrium between the sample and its headspace, and (2) equilibrium between the headspace and the fiber. Because of these independent equilibria, the ratio of any two metabolites in the sample may not be accurately represented on the SPME sorbent, as determined by the activity coefficients of the two compounds [21]. These deviations may arise from intermolecular forces and the interaction between the analyte and the solvent, or other solute molecules.

The first interaction can be estimated from the octanol–water partition coefficient (K_ow_) which describes the hydrophilic or lipophilic nature of the compound [22]. For instance, the equilibrium of very lipophilic metabolites in blood will favor the sample over the headspace due to its increased solubility in the lipid-rich sample [23,24]. Reactions with other solute molecules in the sample represent another factor influencing the first equilibrium. Chief among these in blood is the adsorption of small molecules to proteins present in the sample [25]. Human serum albumin (HSA), the most abundant binding protein in blood, is known to bind with a variety of substances, including fatty acids, bilirubin, drugs, and many other low-molecular-weight molecules [26,27]. As a result, the amounts measured by headspace analysis reflect only the unbound portion of these molecules, not the total amounts present in samples. Biomarker discovery remains challenging due to this complex matrix effect [28].

In this study, we aimed to understand how interactions between volatile metabolites and components of the sample matrix affect detection and quantitation in headspace. Several volatile compounds in different sample media were investigated. Metabolite compounds selected in this study represent common small metabolites in biosamples. Lipid and serum solutions were used to simulate common biological sample conditions in biomarker discovery research. They were chosen in order to provide a range of equilibria between headspace and sample. We also focused on overcoming these forces to reliably employ headspace analysis to make important measurements attributed to sample donors.

## 2. Materials and Methods

### 2.1. Materials

All standard reference materials including 1-hexanol, hexanal, octanal, nonanal, 2-heptenal, 2-octenal, 2,4-octadienal, 2-nonanone, benzaldehyde, and acetophenone-2′,3′,4′,5′,6′-d5 were purchased from Sigma-Aldrich (St. Louis, MO, USA) and the purity of the compounds was between 98.0 and 99.7%. Bovine serum albumin (BSA) and fetal bovine serum were purchased from Sigma-Aldrich and American Type Culture Collection (ATCC; Manassas, VA, USA), respectively. Lipid emulsion was also purchased from Sigma-Aldrich as a product of 20% intralipid.

### 2.2. Methods

#### 2.2.1. Headspace Sampling

A TriPlus RSH autosampler (Thermo Fisher Scientific, Waltham, MA, USA) was used for all analyses. Test solutions (200 µL) were transferred to 20 mL glass vials and the vial was sealed immediately with a magnetic crimp cap. Unless specified, samples were incubated at 40 °C with spinning at 500 rpm for 10 min, followed by 10 min SPME extraction at the same temperature. A 1.10 mm DVB/C-WR/PDMS arrow fiber was used for 10 min extraction at the same temperature of incubation but with a 1000 rpm stirring speed. Following extraction, the fiber was inserted into the injection port of GC and thermally desorbed in splitless mode at 230 °C for 2 min. After the desorption step, the fiber used was baked in the conditioning station to remove all the residual analytes at 260 °C for 10 min.

#### 2.2.2. Analytical Instrument Settings

Chromatographic analyses were conducted using a Thermo Scientific 1300 gas chromatograph (GC) and single quadrapole (ISQ) mass spectrometer (Waltham, MA, USA) equipped with a 30 m × 0.25 mm Stabilwax (1.0 µm film) column (Restek Corp., Bellfonte, PA, USA). The following GC parameters were employed: The oven temperature was held for 2 min at 40 °C and then programmed at 5.5 °C/minute to 230 °C with a 2 min hold at this final temperature. Helium carrier gas was used at a constant flow of 1.1 mL/min. The mass spectrometer was operated at an ionization energy of 70 eV with a 3.3 Hz scan rate over a scan range of *m/z* 33–400 and an ion source temperature of 260 °C. A 7 min solvent delay time was used.

#### 2.2.3. Test Solution Preparation

Ten organic compounds with various functional groups as well as a deuterated internal standard were used to evaluate headspace responses (Table 1, Supplementary Table S1). Test solutions were prepared in water, serum, fat emulsion, and other types of media, respectively. Concentrated solutions of volatile analytes were first prepared by placing 10 mg of each volatile in 1 mL absolute ethanol. Aqueous fortification solutions were prepared by placing ten µL of the concentrated solution in 3.0 mL of water, resulting in a final concentration of 0.03 mg/mL. Samples were prepared by fortifying 1.0 mL of different media, such as water, bovine serum, intralipid, or modified serum solutions with 10 µL of the aqueous fortification solution to yield 0.3 ppm solutions of each analyte. Each solution was subjected to headspace analysis five times.

#### 2.2.4. Effect of Analyte Solubility on Headspace Concentration

In experiment 1, solutions of 1-hexanol, octanal, and 2-nonanone along with the acetophenone-d5 internal standard were prepared in water, fetal bovine serum, or 1% intralipid, respectively. These were selected to offer a range of polarity in both the analytes and the medium. The medium of 1% intralipid was to imitate biological fluids and was prepared by simple dilution of commercial 20% intralipid. Raw and normalized peak responses were separately subjected to two-way analysis of variance (ANOVA) using the Generalized Linear Model (proc GLM) in SAS [30]. Media (water, serum, and intralipid) and volatile compound (1-hexanol, octanal, and 2-nonanone) were fixed effects (acetophenone-d5 was also included in the analysis of raw peak responses). Multiple comparisons of least-square means (Tukey) were made using the ‘pdiff’ option in SAS.

#### 2.2.5. Effect of Matrix Protein on Headspace Concentration

Protein-free serum was prepared by denaturing serum proteins. Two mL of a mixture of acetonitrile, methanol, and acetone (8:1:1) was added to 1.0 mL of serum in a conical centrifuge tube. After mixing for 30 s using a vortex, the solution was centrifuged at 2500 rpm for 5 min and aggregated proteins were removed by decantation. The remaining solution was evaporated until a yellowish residue remained. This residue was diluted with water to the same original volume of 1.0 mL.

In experiment 2, aldehydes were used to compare the various media. A mixture of hexanal, 2-heptenal, octanal, nonanal, and benzaldehyde was prepared at 0.3 ppm concentration in water, serum, and denatured serum and headspace analyses were performed. Water and serum solutions were analyzed three times and denatured serum solutions were analyzed four times. Raw peak response variables were subjected to two-factor ANOVA as in experiment 1.

In experiment 3, solutions with a known protein content were prepared by placing 500 mg of bovine serum albumin in both 10 mL of water and 1% intralipid to match the assumed albumin concentration of serum (5.0 g/dL). Eight volatile compounds representing aldehydes, alcohol, and ketones were tested in a variety of prepared media. Octanal, 2-heptenal, 1-hexanol, 2-nonanone, and 2,4-octadienal were used as analytes together with acetophenone-d5 in water, serum, denatured serum, lipid emulsion, and albumin media to evaluate the impact of sample protein on headspace concentration of these volatiles. Each analysis was repeated five times. Raw and normalized peak responses were subjected to two-factor ANOVA as in experiment 1.

#### 2.2.6. Effect of Temperature on Headspace Concentration

In experiment 4, four volatiles with 8-carbon backbones were chosen to determine the optimal temperature for headspace analysis: 1-octanol, octanal, 2-octenal, and 2,4-octadienal. Test solutions fortified with volatiles (including the internal standard) were prepared in three different media: water, serum, and 1% intralipid. All analyses for extraction optimization were performed in triplicate. The incubation and extraction were carried out for 10 min with a range of temperatures. Each sample was equilibrated at 40, 50, 60, 70, 80, 90, and 100 °C for 10 min of incubation and 10 min of extraction to establish an appropriate equilibrium temperature in each medium condition. The resulting data, consisting of measured headspace peak responses determined at seven different incubation temperatures graphically appeared to fit a second-order polynomial of the type Y = aX^2^ + bX + C. To determine the coefficients (a, b, and C) and estimate critical values (the value of X associated with the highest value of Y), the data were subjected to a quadratic response-surface model (PROC RSREG in SAS). The RSREG procedure uses the method of least squares to fit quadratic functions. For each analyte, the quadratic functions determined for each media type were then compared by analysis of variance (ANOVA) where media type (water, serum, or intralipid solution) was a fixed effect and the parameters of the polynomial function (intercept, coefficient a, and coefficient b) were covariates (PROC GLM). When ANOVA revealed that at least one quadratic function for a volatile differs from the others, linear contrasts were used to investigate which specific media (serum, water, and intralipid) differed from the others.

## 3. Results

### 3.1. Effect of Analyte Solubility on Headspace Concentration

**Figure 1:**
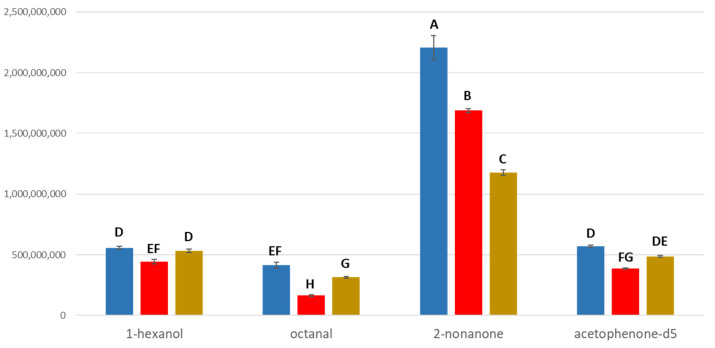
Effect of media solubility on headspace concentration. Analytes are arranged in the order of increasing octanol–water partition coefficients. Headspace responses of analytes were determined in water (blue), serum (red), and 1% intralipid (brown), respectively. Different letters indicate significantly different headspace responses.

The sample matrix effect on volatiles’ headspace concentration was evaluated in experiment 1. Three volatile compounds ranging in octanol–water partition coefficients were added to one of three sample matrices, differing in polarity: 1-hexanol (K_ow_ 1.80), octanal (K_ow_ 2.55), and 2-nonanone (K_ow_ 3.16) in water, serum, or 1% intralipid. There was a highly significant media*volatile interaction (*p* < 0.0001) for the raw responses reflective of compounds that not only differ in media solubility but also differences in response factors arising from electron impact mass spectrometry. In general, the headspace concentrations of volatiles were reduced in lipid-rich serum and intralipid versus polar water (Figure 1). A reduction in HS concentration was correlated with K_ow_ value. 2-Nonanone, being the most lipophilic (as evidenced by having the largest K_ow_ value), exhibited the greatest reduction in peak response among the three odorants in the lipid emulsion relative to water. The HS concentration of 1-hexanol, which is relatively hydrophilic, exhibited the least variation among the three sample matrices, whereas octanal showed the most significant decrease in headspace when analyzed from the serum matrix.

Normalized responses were also subject to media*volatile effects (*p* < 0.0001) (Supplementary Table S2). It was evident that acetophenone-2′,3′,4′,5′,6′-d5 was an appropriate internal standard to account for matrix effects for octanal which showed the greatest variation in the sample matrices. Solubility differences in the three sample matrices became less evident after normalization, as intended. 2-Nonanone, however, failed to be normalized with the assistance of the internal standard due to its high solubility in the lipid medium.

### 3.2. Effect of Matrix Protein on Headspace Concentration

**Figure 2:**
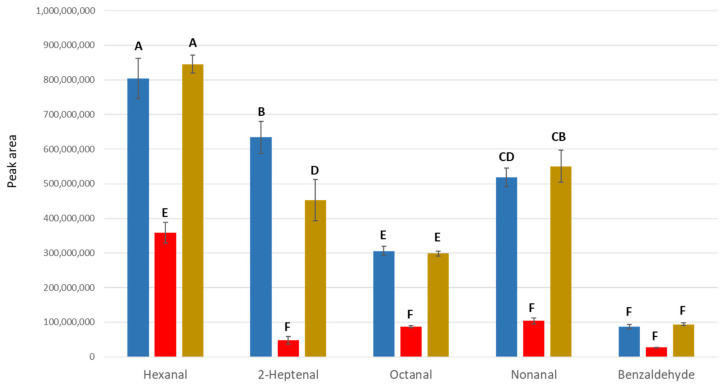
Peak responses of aldehydes observed in modified serum samples. The chromatographic peak responses of several aldehydes were compared in water (blue), serum (red), and modified serum via protein denaturation (brown). Different letters indicate significantly different headspace responses.

After discovering that the HS concentration of octanal decreased significantly in serum, a series of aldehydes were analyzed in water vs. serum in experiment 2. There was similarly a highly significant media*volatile effect in experiment 2 (*p* < 0.0001). Headspace concentrations of aldehydes were consistently lower in serum compared to water, with a more notable reduction seen in unsaturated aldehydes, such as 2-heptenal (Figure 2). Blood proteins were considered to be the primary source of unsaturated aldehyde binding. To investigate likely intermolecular interactions between reactive aldehydes and dissolved solutes in blood serum responsible for this observation, reduced-protein serum was prepared by denaturing and centrifugation. Following protein denaturation and centrifugation, the headspace concentrations of aldehydes from protein-free serum were similar to water solutions (Figure 2).

In experiment 3, the role of proteins in reducing headspace concentrations of volatiles was further confirmed by adding bovine serum albumin (BSA) to both water and intralipid. Several volatile compounds with varying octanol–water partition coefficients were examined in these new sample matrices. In addition, the effect of peak normalization with the internal standard (acetophenone-d5) was also investigated.

**Figure 3:**
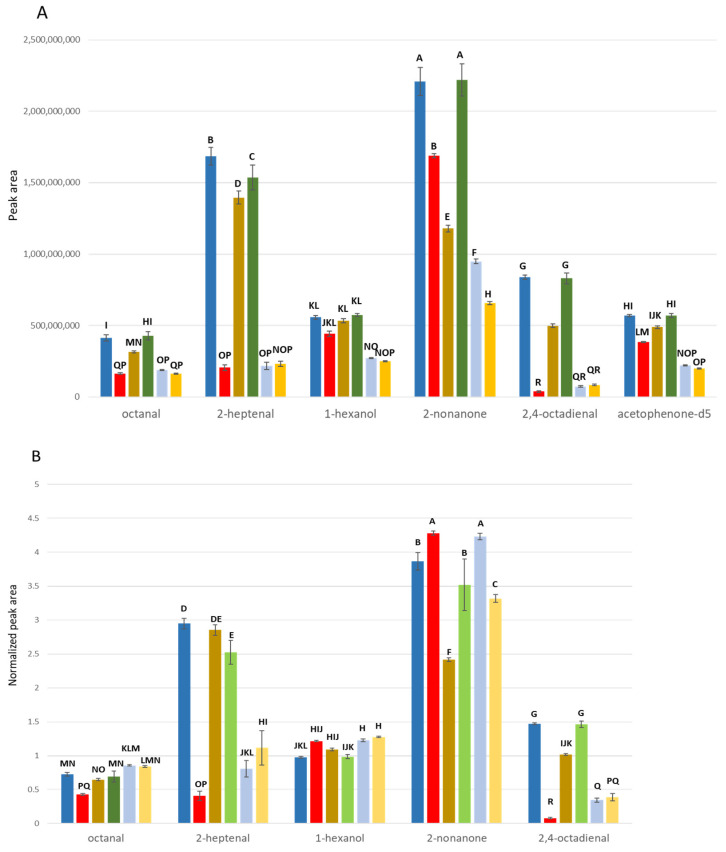
Raw vs. normalized peak responses of several volatiles in various sample matrices. (**A**) Raw peak responses observed in different media of water (blue), serum (red), 1% intralipid (brown), denatured serum (green), albumin solution in water (light blue), and albumin solution in intralipid (yellow). Different letters indicate significantly different headspace responses. (**B**) Peak responses normalized to the internal standard acetophenone-d5 were also calculated in the same medium series. Different letters indicate significantly different normalized headspace responses.

Both raw and normalized responses were subject to the interaction of volatile and media (*p* < 0.0001). Headspace concentrations were suppressed by BSA to the similar extent observed in blood serum (Figure 3). HS reduction from serum correlated with the number of unsaturated bonds in the aldehyde molecules. Conjugated 2,4-octadienal showed the greatest reduction when it was analyzed in serum sample. 2-Nonanone showed the greatest reduction in the lipid solution and this reduction became even more significant with the albumin-fortified lipid. Although protein-induced HS variation was minimized by the internal standard, the internal standard (acetophenone-d5, K_ow_ = 1.65) was clearly not subject to the same protein interactions as the aldehydes. Normalization minimized HS variation among matrices best when the partition coefficients of the internal standard and the analyte (e.g., 1-hexanol, K_ow_ = 1.80) were matched.

### 3.3. Effect of Temperature on Headspace Concentration

**Figure 4:**
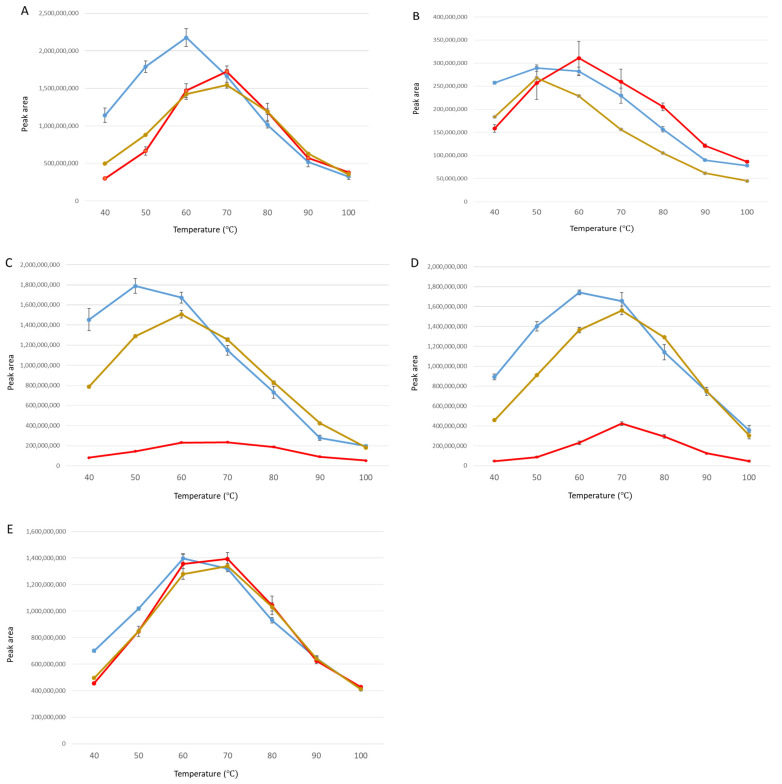
Temperature effect on headspace concentration of compounds. SPME incubation and extraction temperature was manipulated to find the optimum temperature for several volatile compounds of (**A**) 1-octanol; (**B**) octanal; (**C**) 2-octenal; (**D**) 2,4-octadienal; (**E**) acetophenone-2′,3′4′,5′,6′-d5. Different critical temperatures (Table 3) were revealed according to different media of water (blue), serum (red), and intralipid (brown).

In experiment 4, incubation and extraction temperatures ranging from 40 to 100 °C were employed to drive the equilibrium toward the headspace. In general, headspace concentrations from water, serum, and intralipid fit a second-order polynomial function (Figure 4). The headspace responses increased as the temperature approached the critical value (Table 2). Critical values (incubation temperature corresponding to maximum peak response) were generally lowest in water and highest in serum, reflecting differing solubilities. The temperature profiles (i.e., polynomial functions) of the aldehydes were most affected by changes in media. ANOVA results indicated that all three polynomial parameters were impacted by media (serum, intralipid, or water) for the three aldehydes (Table 3). For the alcohol, only the intercept was subject to media effects, whereas none of the polynomial functions arising for the acetophenone-d5 temperature response differed among the media types. Escaped volatiles from the vials under high pressure caused by the elevated temperature likely contributed to the observed polynomial functions (reducing headspace responses at temperatures greater than the critical value).

## 4. Discussion

Chemical analysis of volatiles from biological samples can provide useful insights into metabolic processes and may serve as biomarkers to monitor diseases or health status. Headspace analysis has a distinct advantage of minimal sample preparation for this purpose. However, quantitative analysis relies upon the efficient transfer of volatiles from the sample into the gas chromatograph. Not only are headspace sampling approaches inefficient for compounds with low vapor pressure, but headspace sampling can also be poorly quantitative because volatile collection is subject to multiple equilibria. Thus, without correction, concentrations observed in the headspace may not accurately reflect concentrations present in the sample. This study represents the effort to demonstrate sample matrix effects on headspace concentrations of volatiles and to overcome this to achieve more accurate analyses.

Significant reductions in the headspace responses of volatiles with higher K_ow_ values were observed when the sample matrix was less polar. For example, the headspace concentrations of 2-nonanone (K_ow_ = 3.16) were significantly lower in intralipid versus water (Figure 1). In general, headspace concentrations of all volatiles from serum were reduced in comparison to water. These differences could be mediated by normalization to the internal standard in some cases. However, while acetophenone-2′,3′,4′,5′,6′-d5 accounted for solubility differences in octanal, it failed to normalize the 2-nonanone response in the lipid sample due to its great affinity for lipids. Therefore, in some cases, multiple internal standards might be required for improving quantification in analyses [31]. While salting-out may enhance the efficiency of headspace analysis, its effect also varies between compounds and it does not eliminate matrix effects, potentially causing inaccurate results when dealing with highly complex matrices or analytes with similar properties to matrix [32,33].

Secondly, large reductions in headspace concentrations may result from the strong interaction between the volatile molecules and other solutes in the sample matrix. It was found that headspace concentrations of aldehydes were significantly reduced from the serum sample matrix (Figure 2). Aldehydes, produced by a lipid peroxidation, easily react with proteins in blood via a wide variety of intra- and inter-molecular covalent adducts. They can form Schiff bases and/or Michael adducts with the free amine group of lysine, the imidazole group of histidine, the guanidine group of arginine, and thiol group of cysteine [34]. This interaction can significantly alter aldehyde properties, affecting extraction efficiency for these important metabolites. Relative to water, the headspace concentration of the aldehydes in other matrices increased when proteins were removed (Figure 2). Similarly, the addition of a protein (BSA) reduced the headspace concentration of several aldehydes due to these same intermolecular forces (Figure 3). Together, these results revealed that the proteins were the main source of the interactions with the volatiles and resulted in reduction in headspace concentration. Although a significant number of metabolite–protein interactions have already been discovered, only a small portion of interaction networks have been identified [35]. Unraveling the interaction networks by systematic approach can further help to understand the molecular basis of healthy and diseased states and promote the availability of unbound volatile metabolites in headspace analyses.

One way of improving the sensitivity of the headspace analysis is to raise the temperature of the sample to drive the equilibrium closer to the vapor phase [36]. It was also anticipated that the interactive forces between volatiles and media might become negligible at higher temperatures. Elevated temperatures from 40 to 100 °C were evaluated (Figure 4). Critical values (temperature associated with highest headspace concentration) widely varied in water (Table 3) and were elevated in serum. Even though the factors controlling equilibrium in headspace analyses may be very complex, an incubation and extraction temperature of 70 °C appears to be the optimal extraction temperature for volatile metabolites in biological samples.

In conclusion, the volatile amounts detected in the headspace from the samples were notably affected by the sample matrix, and the deviations were determined not only sample matrix, but also structural properties of the volatile molecules. The solubility of an analyte in the matrix played a key role in determining its mass transfer into the headspace. The greater the solubility, the less likely the analyte was to partition into the headspace, resulting in lower headspace responses by GC-MS. A significant reduction in headspace concentration was noted for aldehydes, more specifically conjugated aldehydes. One challenge in quantifying reactive aldehydes in biosamples would be the possible requirement of additional sample processing [37]. The matrix effects may be overcome by increasing the extraction temperature to inactivate blood proteins in a HS-SPME procedure. The optimized extraction temperature of 70 °C benefitted recovery of these important oxygenated volatiles. Overall, these results further demonstrate the need for proper method development and validation. It is essential to emphasize that no one set of conditions can be considered sufficient for broad applications in biomarker discovery.

## Figures and Tables

**Table 1:** List of volatile compounds used for sample preparation and their Log K_ow_.

Volatile Compound	Log K_ow_ [29]
1-Hexanol	1.80
Hexanal	1.78
2-Heptanal	2.06
Octanal	2.55
2-Octenal	2.57
2,4-Octadienal	2.35
Nonanal	3.27
2-Nonanone	3.16
Benzaldehyde	1.48
Acetophenone	1.65

**Table 2:** ANOVA probabilities for analysis of media effects (serum, intralipid, or water) on the polynomial terms describing the relationship between incubation/extraction temperature and headspace concentration. The second-order function follows the form Y = aX^2^ + bX + C.

Volatile	a-Term	b-Term	Intercept (C)
1-Octanol	0.3676	0.1251	0.0101
Octanal	0.0033	0.0012	0.0009
2-Octenal	<0.0001	<0.0001	0.0001
2,4-Octadienal	<0.0001	<0.0001	<0.0001
Acetophenone-2′,3′,4′,5′,6′-d5	0.2471	0.1535	0.0749

**Table 3:** Critical values (incubation/extraction temperature associated with maximum headspace concentration) for each volatile in serum, intralipid, and water media.

Volatile	Log K_ow_	Serum	Intralipid	Water
1-Octanol	N/A	69.7 °C	68.2 °C	59.6 °C
Octanal	2.55	63.5 °C	40.5 °C	41.4 °C
2-Octenal	2.57	67.6 °C	61.1 °C	39.0 °C
2,4-Octadienal	2.35	70.7 °C	68.7 °C	64.1 °C
Acetophenone-2′,3′,4′,5′,6′-d5	1.65	68.5 °C	68.2 °C	65.3 °C

## Data Availability

The data presented in this study are available from the corresponding author upon request.

## Supplementary Materials

The following supporting information can be downloaded at: https://www.mdpi.com/article/10.3390/metabo15030183/s1, Table S1: Volatile compounds mixture and medium analyzed in this study; Table S2: Chromatographic peak area of volatile compounds in different media; Table S3: Chromatographic peak area of several aldehydes in different media; Table S4: Chromatographic peak area of several volatile compounds in different media; Table S5: Chromatographic peak area of several volatiles in different temperatures.
